# Anti-Inflammatory Effects of Polyglycerol Sulfates and Natural Polyanions in Type 2 Inflammation

**DOI:** 10.1021/acs.biomac.5c00420

**Published:** 2025-05-23

**Authors:** Clemens Krage, Peyman Malek Mohammadi Nouri, Jens Dernedde, Jayachandran N. Kizhakkedathu, Sarah Hedtrich, Rainer Haag, Katharina Achazi

**Affiliations:** † Institute für Chemie und Biochemie, 9166 Freie Universität Berlin, Takustrasse 3, D-14195 Berlin, Germany; ‡ Centre for Blood Research, Life Sciences Institute, 461705 University of British Columbia, 2350 Health Sciences Mall, Life Sciences Centre, Vancouver, British Columbia V6T 1Z3, Canada; § Institut für Laboratoriumsmedizin, klinische Chemie und Pathobiochemie, Charité-Universitätsmedizin Berlin, Augustenburger Platz 1, 13353 Berlin, Germany; ∥ Department of Pathology and Laboratory Medicine, University of British Columbia, 2350 Health Sciences Mall, Life Sciences Centre, Vancouver, British Columbia V6T 1Z3, Canada; ⊥ The School of Biomedical Engineering, University of British Columbia, 2350 Health Sciences Mall, Life Sciences Centre, Vancouver, British Columbia V6T 1Z3, Canada; # Faculty of Pharmaceutical Sciences, University of British Columbia, Vancouver, British Columbia V6T 1Z3, Canada; ∇ Berlin Institute of Health at Charité−Universitätsmedizin Berlin, 10117 Berlin, Germany

## Abstract

Type 2 inflammation is an essential defense mechanism of the innate and adaptive immune systems, but when dysregulated, it can cause chronic atopic diseases like allergic asthma and atopic dermatitis. Thymic stromal lymphopoietin (TSLP) helps drive type 2 inflammation by guiding T cells toward a type 2 helper cell (T_H_2) subtype and stimulating B cells’ antibody production. Fibronectin (FN) has recently been found at elevated levels in the plasma of children with atopic dermatitis and shown a potential proinflammatory role in bronchial epithelium tissue models. Both proteins’ surface charges suggest potential interaction with charged molecules. Seeking new strategies against type 2 inflammation, we found that negatively charged polyglycerol sulfates strongly bind to TSLP and FN. We confirmed that these molecules inhibit inflammation by reducing the TSLP-mediated type 2 polarization of CD4^+^ T cells. We found that adding polyglycerol sulfate to FN-triggered inflamed bronchial epithelium models reduced TSLP expression and interleukin 6 secretion.

## Graphical abstract

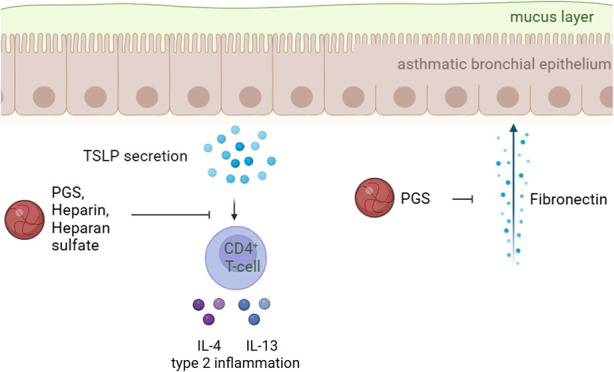

## Introduction

Type 2 inflammation is driven by the adaptive and innate immune systems. It plays a central role in defending against pathogens by activating type 2 helper cells (T_H_2) and type 2 innate lymphoid cells (ILC2), which drive inflammation through the production of type 2 cytokines such as interleukin (IL)-4 and IL-13. While type 2 inflammation is an essential weapon in protecting against pathogens, its dysregulation can lead to chronic damage of the epithelium. Dysregulated type 2 inflammation manifests as a variety of common epithelial diseases, such as atopic dermatitis (AD), allergic asthma (AA) and eosinophilic esophagitis.^1–3^ The observed correlation of AD and AA led to the hypothesis of the “atopic march”, which refers to the progression from AD in infants to AA in children and adults, with both conditions caused by type 2 inflammation. Recent studies suggest that this progression may result from an interepithelial crosstalk between the skin and the lungs.^4,5^

The cytokine thymic stromal lymphopoietin (TSLP), a known driver of type 2 inflammation, has been identified as a major contributor to the atopic march. TSLP is an IL-7-like cytokine involved in the activation and maturation of dendritic cells as well as the direct activation of CD4^+^ T cells. TSLP plays an essential role in the cascade of immune responses that generate type 2 inflammation, whether in response to a harmful parasite or in response to an otherwise harmless allergen. Epithelial cells such as keratinocytes in the skin and lungs produce TSLP, then release it in response to a perceived threat.^6–8^ TSLP interacts with the TSLP receptor (TSLPR) via electrostatic interactions, forming a complex that subsequently binds to IL-7Rα.^9^ This binding process cannot proceed without a charge–charge interaction between positively charged patches of TSLP and TSLPR. This trimeric complex then induces IL-4 and IL-13 release via STAT5 and STAT6 phosphorylation in CD4^+^ T cells.^9–13^ In turn, these type 2 cytokines stimulate antibody production by B cells as well as the expansion of basophils and eosinophils in the epithelium; the result is type 2 inflammation.^4,14^ Further effects are the IL-13-mediated stimulation of goblet cells in bronchial epithelia, resulting in elevated expression of mucin 5 AC (MUC5AC) and thereby leading to mucosal plugs.^15^

The increasing prevalence of atopic diseases, along with TSLP’s position at the top of the inflammatory cascade, render it an attractive drug target.^16–19^ Initial attempts at blocking the TSLP–TSLPR interaction by injection of soluble TSLPR showed a promising reduction of type 2 cytokines and reduced the infiltration of eosinophils and lymphocytes into the bronchiolar lavage fluid in mice.^20^ A more advanced approach, based on developing TSLPR-IL7Rα fusion proteins as cytokine traps, was shown to potently reduce the TSLP-mediated maturation of dendritic cells.^9^ The subsequent development of targeted antibodies led to the recent regulatory approval of the TSLP antibody Tezepelumab for the treatment of AA.^21,22^ However, alternate drugs are still desirable due to the general drawbacks of antibody therapies: high cost, tolerance buildup, and the likelihood of allergic reaction.

This high demand for alternate therapies recently led to the discovery of the small molecule TSLPR- inhibitor BP79 and Baicalein as a TSLP inhibitor.^11,23^ BP79, alongside with structurally related TSLPR inhibitors, was found to significantly reduce the TSLP-mediated T_H_2 response of CD4^+^ T cells, and it reduced immune cell infiltration in multiorgan models. Baicalein inhibited STAT5 phosphorylation in mast-cell lines after TSLP stimulation and reduced eosinophil infiltration in ovalbumin-challenged mouse models. These results show the importance of better understanding the TSLP–TSLPR interaction to find new treatment options.

Recent findings of elevated plasma fibronectin (FN) levels in infants with atopic diseases also led to the observation that FN can increase TSLP expression in bronchial epithelium tissue models.^24^ The discovery of FN as a potential contributor to epithelial inflammation establishes FN interception as a potential new route for inhibiting type 2 inflammation.

FN displays large positively charged patches on its surface, suggesting interaction potential with negatively charged molecules, such as sulfated polyglycerols (PG) and natural polyanions like heparin and the structurally related heparan sulfate (HS).^25^ This interaction potential also applies to TSLP, since its binding with TSLPR relies upon electrostatic interaction with negative patches on TSLPR.^4,9^ The significance of these positive charges for the function of both proteins led us to hypothesize that highly charged molecules may interfere with the binding mechanisms that enable these proteins’ roles in the inflammatory cascade.

The concept of disrupting signaling pathways by electrostatic interactions using highly negative charged molecules has been applied before: dendritic polyglycerol sulfate (dPGS) was first confirmed to inhibit inflammation by electrostatically interacting with P- and L-selectin, thus reducing leukocyte extravasation.^26–28^ Later, interaction with complement system proteins C1q, C3a and C5a was observed, leading to complement activation reduction via all three pathways.^29^ Anti-inflammatory effects have also been observed for cationic polymers: polyamidoamine dendrimers were shown to interact with cyclooxygenase 2 and reduce inflammation in vivo.^30,31^

A clinical application that follows this principle is the use of heparin to reduce blood clot formation. Heparin is a highly negatively charged sulfated and carboxylated glycosaminoglycan. It binds to antithrombin III (ATIII), which then in turn can inactivate thrombin (Factor IIa) and Factor Xa, two key coagulation proteins involved in the blood clotting process.^32,33^ Heparin is also known to possess anti-inflammatory effects, such as its interactions with the complement pathway, its ability to reduce neutrophil adhesion to the endothelium, and its possible interaction with NF-κB.^34–36^ However, the potent anticoagulant effect of heparin prevents its use as a therapeutic agent for anti-inflammatory purposes due to the danger of increased bleeding as a side effect.^37^ HS is structurally related to heparin but exhibits lower anticoagulant activity. Both polysaccharides consist of disaccharide units consisting of modified uronic acid β-1 → 4 linked to *N*-acetylglucosamine that are connected via an α1 → 4 linkage. While glucuronic acid is the predominant uronic acid epimer in heparan sulfate, it is largely epimerized to iduronic acid in heparin, which is generally considered the more extensively modified form of HS.^33^

In this study, we investigated the potential use of sulfated or carboxylated polyglycerols (dendritic and linear versions) as heparin analogues and analyzed their binding to proinflammatory proteins such as TSLP and FN via surface plasmon resonance spectroscopy (SPR). In this preliminary analysis we sought a better understanding of how binding would be influenced by charged groups, the difference between carboxyl and sulfate groups, and polymer architecture. We then aimed to build on the SPR results to assess the investigated polyglycerols’ potential as anti-inflammatory agents in type 2 inflammation.

We used an inflamed three-dimensional (3D) bronchial epithelial tissue model as well as a T_H_2-polarization model of CD4^+^ T cells to investigate the effect of a library of polyanion candidates on type 2 inflammation.

## Materials and Methods

### Synthesis of a Polyanion Library

The detailed synthesis of polyglycerol (PG) as well as its functionalization have been described previously (SI Figure 1).^38–42^ Sulfation of dendritic and linear precursor molecules (dPG and lPG) was achieved with sulfamic acid and gave the corresponding polysulfates dPGS and lPGS. For carboxylation, succinic anhydride was added to linear or dendritic PG. All products were purified by dialysis. The degree of sulfation was determined via elemental analysis and the degree of carboxylation by NMR spectroscopy (SI Figures S2–S5). Compounds dPGS_50(90%)_ and dPGS_100(90%)_ were prepared as published previously and contained 3.3% caprolactone units in the polymer backbone.^43^

### Surface Plasmon Resonance (SPR) Spectroscopy Analysis

Binding experiments were performed on a BIAcoreX100 device (GE Healthcare) according to the manufactures’ protocols.

In brief, recombinant biotinylated TSLP (Sino Biological) was immobilized on a standard streptavidin coated (SA) biosensor (GE Healthcare) until ∼50 resonance units (RU) were reached. As soluble analytes, serial dilutions of polyanions were subsequently injected for 150 s, followed by a dissociation time of 600 s and a 30 s injection of 2 M NaCl to regenerate the surface. All SPR measurements were conducted in standard HBS-EP buffer (150 mM NaCl, 10 mM HEPES, 3 mM EDTA, 0,05% P20, pH 7.4). The chemicals NaCl, HEPES and EDTA were purchased from Carl Roth. The binding affinities were determined by using the BIAcore evaluation software version 4.0 using a 1:1 kinetic fit. The reference lane was left unmodified.

The binding to FN was determined by an inhibition assay. Here, biotinylated heparin (Merck) was injected to yield ∼300 RU. The reference was coated with biotinylated lPG as a control surface imitating the linear uncharged heparin architecture. Then, 250 nM FN or 250 nM FN preincubated with the respective polyanion concentration was injected. The response of 250 nM FN was taken as the 100% value. Again, all SPR measurements were conducted in standard HBS-EP buffer. IC_50_ values were determined by using a logistic fit in Origin 2022 by plotting the response level in % of the control after 178 s (2 s before starting the dissociation) versus the inhibitor concentration (SI Figure 9).

### Toxicity Analysis

Fresh human CD4^+^ T cells were isolated from human blood at the Centre for Blood Research, University of British Columbia (UBC) according to the standard protocol using EasySep Direct Human CD4^+^ T cell isolation kit (STEMCELL). The blood collection protocol was approved by the UBC clinical ethical board (H20–00084) and the blood was collected from consented donors. 100,000 Human CD4^+^ T cells per well were cultured in 100 μL RPMI 1640 (ThermoFisher) supplemented with 10% fetal bovine serum (FBS) (ThermoFisher) and 1% Penicillin/Streptomycin (P/S) (ThermoFisher). After 24 h, polyanions were added at three different concentrations in triplicates. Further 24 h later, cell toxicity was analyzed with the cell counting kit CCK-8 (Merck) according to the manufacturer’s description.

### T Cell Activation Studies

Human primary CD4^+^ T cells were cultivated in RPMI 1640 + 10% FBS + 1% P/S. The cells were seeded at 80,000 cells per well in 100 μL in a 96 well plate and then activated for cytokine secretion with a cell stimulation cocktail (PMA 100 ng/mL; ionomycin 1 μg/mL) from ThermoFisher and 50 ng/mL TSLP (PeproTech). Polyanions as potential inhibitors of activation were then added to the wells and IL-13 levels were determined after 36 h by ELISA as a readout. To determine the difference in STAT6 phosphorylation state, cells were lyzed 20 min after adding the cytokines and inhibitors. For the investigation of the polyanion’s TSLP-specific effects, human primary CD4^+^ T cells were seeded at 100,000 cells per well in 100 μL in a 96 well plate and then activated for cytokine secretion with a cell stimulation cocktail (PMA 100 ng/mL; ionomycin 1 μg/mL) (ThermoFisher) or the CD3/CD28-antibody containing Dynabeads human T-cell activator (ThermoFisher). The cells were incubated for 72 h as recommended in the manual and the supernatant was investigated for its IL-13 concentration.

### ELISA

CD4^+^ T cell culture supernatants were first centrifuged at 500*g* for 5 min to remove cells and then at 13,000*g* for 5 min to remove cell debris. ELISAs for IL-13 (Invitrogen) as well as IL-6 (Proteintech) and IL-8 (Proteintech) were performed according to the manufacturer’s instructions. For the screening of dPGS inhibition of TSLP–TSLPR-IL7Rα interaction, IL7Rα & TSLPR Inhibitor Screening ELISA Kit was purchased by ACROS biosystems and performed according to the manufacturer’s instructions.

### Western Blot Analysis

CD4^+^ T cells were centrifuged for 5 min at 500*g* to remove the supernatant and then washed with ice-cold PBS. The PBS was then removed and the cells were lysed on ice for 30 min using freshly prepared RIPA-Buffer (ThermoFisher) containing Protease/Phosphatase inhibitor (ThermoFisher). Protein concentrations were determined using a BCA-Assay (ThermoFisher). After adding Laemmli buffer and heating to 95 °C for 5 min 10 μg of total protein lysates were applied on a 4 to 20% precast gel (BIO-RAD). After electrophoresis, the proteins were transferred to a PVDF membrane (BIO-RAD). After 1 h of blocking with 5% nonfat milk (sigma) in TBST (20 mM Tris, 150 mM NaCl, 0.1% Tween) primary antibodies (STAT6: D3H4,Cell signaling technologies, P-STAT6: D8S9Y, Cell signaling technologies, β-Actin: ab8224, abcam, TSLP: ab4739, abcam) were added at 1 μg/mL in 5% milk in TBST and incubated overnight at 4 °C. After washing, secondary antibodies (IR-Dye 680RD Donkey anti mouse IgG, IR-Dye 680RD goat anti rabbit IgG (Fisher Scientific)) were incubated for 1 h at room temperature. After washing, blots were imaged on an Odyssey imaging device. The representative uncropped Western Blots are displayed in the SI (SI Figure 10).

### Generation of 3D Bronchial Epithelial Tissue Models

Bronchial epithelial tissue models were generated as described before.^44^ Briefly, 6.4 × 10^4^ human bronchial fibroblasts were mixed with FBS and bovine collagen I (PureCol, Cedarlane), adjusted to neutral pH and added onto a 12 well cell culture insert. After 2 h of CO_2_-free incubation at 37 °C, expansion medium (PneumacultExPlus, STEMCELL) was added into the well and onto the inset. After additional 2 h of incubation at 37 °C, 5% CO_2_, the media on the inset was removed and 0.9 × 10^6^ human bronchial epithelial cells were added. After 24 h, the media in the well was replaced by differentiation medium (Pneumacult ALI media, STEMCELL) and the media in the inset was removed. Then, every second day, media was changed. At day 16, 18, and 20, 1 μg/mL human plasma derived fibronectin (ThermoFisher) was added to trigger an inflammation. At day 18 and 20, dPGS_10(90%)_ was added to the treated wells. The models were harvested at day 21. For Western blot analyses, the models were lyzed in a Tissuelyser II (Qiagen) with beads at 25 Hz for 5 min.

### Immunofluorescence Staining

Models were placed in tissue freezing media (Leica Biosystems) and frozen at minus 80 °C. Then, 9 μm slices were cut and placed on a poly-l-lysine coated microscopy slide. The sections were then washed with PBS and permeabilized with Triton X. After washing, blocking was performed with 1:20 diluted goat serum (ThermoFisher) in PBS for 30 min at room temperature (RT). Next, the primary antibody (Abcam, ab47943) was added for 1 h. After washing with PBS, the secondary antibody was applied for 1 h and after further washing steps, slides were dried and mounted with 4,6-diamidino-2-phenylindole (DAPI)-containing Fluoroshield medium (Sigma). Overnight incubation was performed at 4 °C before imaging on a fluorescence microscope (Keyence BZ-X810).

### Statistical Analysis

All statistical analysis was performed with GraphPad Prism 9. Values are expressed as means ± SD. For a sample size of *n* = 3, Friedman test was performed. For *n* = 9, one-way analysis of variance (ANOVA) test was performed. The specific statistical test is described in the individual figure caption. *P* value ≤ 0.05 was considered as statistically significant.

## Results and Discussion

### Synthesizing the Polyanion Library

For the synthesis of the polyanion library, we chose linear as well as dendritic polyglycerol as polymer backbones. Polyglycerol is bioinert, and the required negatively charged sulfate or carboxylate groups can be introduced via the free hydroxyls (Scheme 1A–C).^45,46^ We successfully functionalized a linear and a dendritic version of the polyglycerol with similar sizes (∼5 kDa before and ∼10 kDa after 90% functionalization) with either sulfate or carboxylate groups with a degree of functionalization around 90%. This enabled us to determine how the specific functional group and backbone flexibility affected binding and modulation of inflammation. We also functionalized dendritic polyglycerol with different amounts of sulfate groups (40, 70, 90%) to assess the effect of increased charge density. Further, two larger polyglycerol sulfates were synthesized with final molecular weights of ∼50 and ∼100 kDa to determine the effect of the polyanion size. Table 1 provides an overview of the synthesized linear and dendritic polyglycerol sulfates and carboxylates as well as the results regarding their size, degree of functionalization, number of anionic groups and ζ-potential. The corresponding NMR spectra are provided in the SI (SI Figures S1–S5).

**Scheme 1:**
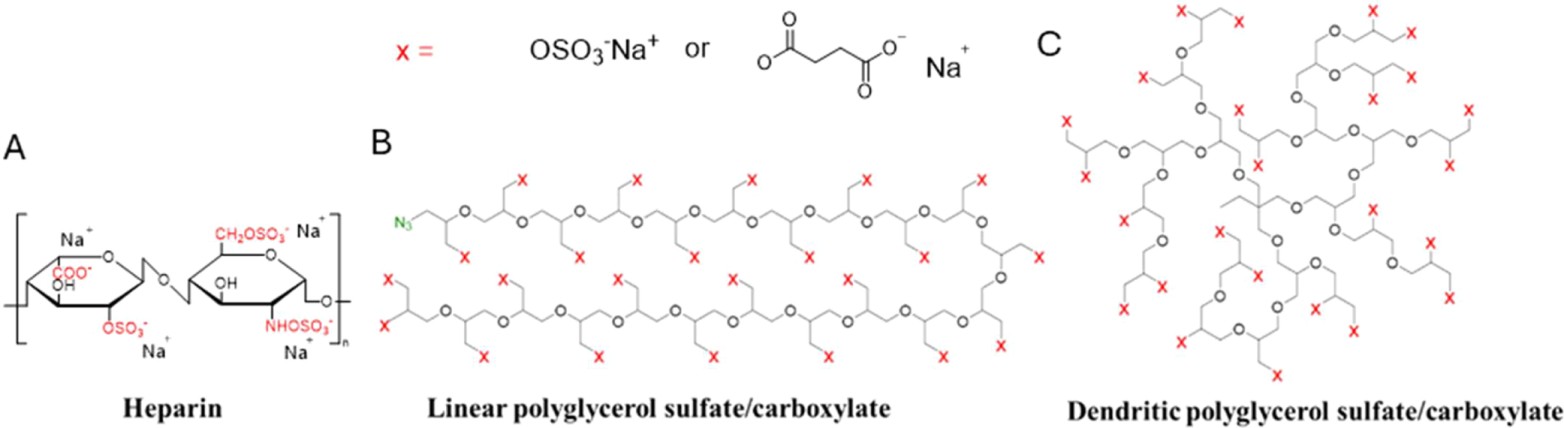
Structure of (A) Heparin, (B) Linear Polyglycerol Sulfate and Carboxylate, and (C) Dendritic Polyglycerol Sulfate and Carboxylate

**Table 1:** Size, Degree of Functionalization, and Charge of Synthesized Polymers

polyanion	*M_n_* (kDa)^a^	dF (%)^b^	# of anionic groups	ζ (mV)^c^	*d* (nm)^d^
dPG	5	0	0	–12 ± 3	5 ± 1
lPG	5	0	0	–15 ± 1	4 ± 1
dPGS_7(40%)_	7.2	40	27	–26 ± 3	5 ± 2
dPGS_9(70%)_	8.8	70	47	–32 ± 3	5 ± 1
dPGS_10(90%)_	9.9	91	62	–37 ± 2	5 ± 2
dPGS_50(90%)_	49.3	90	304	–39 ± 1	11 ± 3
dPGS_100(90%)_	98.7	90	608	–29 ± 1	15 ± 3
lPGS_10(90%)_	9.9	88	60	–33 ± 4	4 ± 1
dPGC_10(90%)_	11.1	80	54	–26 ± 1	6 ± 1
lPGC_10(90%)_	11.5	96	65	–33 ± 1	5 ± 1

a Molecular weight (*M_n_*) was determined by gel permeation chromatography (GPC) for the unsulfated compounds and calculated according to the functionalization degree for the polyanions.

b Degree of functionalization (dF) was determined by elemental analysis for sulfates and ^1^H NMR for carboxylates.

c Zeta potential (ζ) was measured in 10 mM phosphate buffer (pH = 7.4)

d Hydrodynamic diameter (*d*) was measured by dynamic light scattering (DLS) in PBS.

### Investigating Polyanions’ Binding to TSLP by SPR

For an initial assessment of the influence of charge, size, flexibility and anionic group, the synthesized polyanions were first analyzed regarding their binding to TSLP. These results were compared to those observed with heparin and heparan sulfate (HS) using SPR. The synthetic polyglycerol sulfates showed remarkably strong binding to TSLP, outperforming the natural polymers heparin and heparan sulfate (Figure 1A–F). The calculated binding constants are shown in Table 2. Here, linear polyglycerol sulfate (lPGS_10(90%)_) showed the strongest binding with an equilibrium dissociation constant of *K*_D_ = 0.565 ng/mL, while the dendritic variant with the same size of 9.9 kDa and equivalent degree of sulfation (90%) showed a 5-fold less affine binding with an equilibrium dissociation constant of *K*_D_ = 2.9 ng/mL. Thus, the linear PG architecture is preferred, with the flexible presentation of sulfate groups allowing for more efficient charge–charge interactions. Reducing the degree of sulfation from 90 to 70% only led to a slight reduction of affinity (SI Figure 6), while a reduction to 40% sulfation resulted in no binding. Further experiments also confirmed the ability of dPGS_10(90%)_ to block TSLP from interaction with a fusion protein of the receptor complex TSLPR and IL7Rα (SI Figure 7). Here, 711 ng/mL were necessary to reduce the binding of TSLP to the receptor complex fusion protein by 50%. This relatively high concentration in comparison to the *K*_D_ of dPGS_10(90%)_ to TSLP can be explained by the extraordinarily high affinity binding to the fusion protein; previous literature describes a *K*_D_ of TSLP to TSLPR-IL7Rα fusion proteins of *K*_D_ = 60–120 pM. In physiological conditions, however, TSLP first associates to TSLPR (*K*_D_ = 32 nM) and this complex then binds IL7Rα (*K*_D_ = 29 nM).^9^ Thus, an interaction blocking the initial interaction of TSLP with TSLPR would most likely occur at markedly reduced concentrations compared to those necessary to block the interaction of TSLP with a TSLPR-IL7Rα fusion protein.

**Table 2:** Binding Constants of Functionalized and Natural Polymers to TSLP

polyanion	*M_n_* (kDa) weight (kDa)	*K*_D_ (pM)^a^	*K*_D_ (ng/mL)	*k*_a_ (1/Ms)^b^	*k*_d_ (1/s)^c^
dPGS_7(40%)_	7.0	no binding			
dPGS_9(70%)_	9	404	3.6	1.17 × 10^6^	4.74 × 10^–4^
dPGS_10(90%)_	9.9	290	2.9	1.49 × 10^6^	4.32 × 10^–4^
lPGS_10(90%)_	9.9	56.5	0.565	1.49 × 10^7^	8.41 × 10^–4^
dPGS_50(90%)_	49.3	38.6	1.93	5.45 × 10^6^	2.10 × 10^–4^
dPGS_100(90%)_	98.7	124.7	12.5	2.96 × 10^6^	3.69 × 10^–4^
heparin	15	1 100	16.5	1.05 × 10^6^	1.19 × 10^–3^
heparan sulfate	40	87 000	3 480	1.62 × 10^3^	1.42 × 10^–4^
dPG	5	no binding			
dPGC_10(90%)_	11.1	no binding			
lPGC_10(90%)_	11.5	no binding			

a Equilibrium dissociation constant (*K*_D_)

b Association constant (*k*_a_)

c Dissociation constant (*k*_d_)

**Figure 1:**
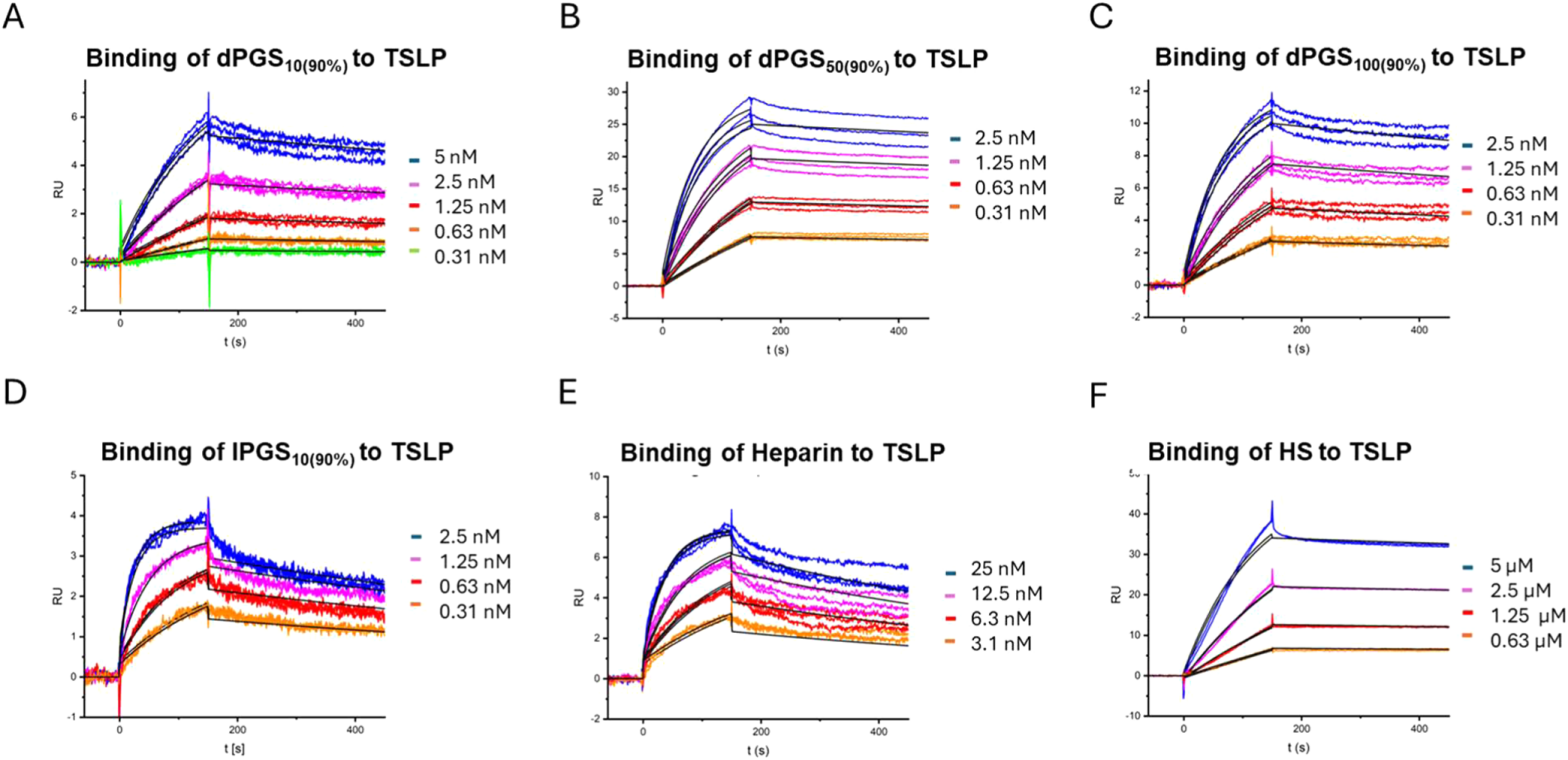
(A–F) SPR sensorgram binding profiles. Measurements were performed in standard HBS-EP buffer at 25 °C. Biotinylated TSLP was immobilized on a streptavidin (SA) chip, and then polyanion dilutions were injected. The 1:1 global kinetic fit curves given by the BIAevaluation software are shown as black lines.

In contrast to the polyglycerol sulfates, the polyglycerol carboxylates did not bind to TSLP. This effect has been observed previously with other proteins, suggesting that sulfates play a unique role in the binding process. When investigating the binding affinity of differently functionalized polyglycerols to l-selectin, we found that the interaction strength increased in the following order: carboxylate < phosphate < phosphonate = sulfonate < bisphosphonate < sulfate.^27^ This difference might result from variations in the polyanion protonation degree, since polycarboxylates have higher p*K*_a_ values than polysulfates.^47^ This hypothesized effect did not lead to reduced ζ-potentials of the carboxylates compared to the sulfates. This can be attributed to the fact that the ζ-potential only describes the potential difference at the slipping plane rather than the direct surface of the polymer. We also observed a strong interaction of heparin with TSLP (*K*_D_ = 16.5 ng/mL), while HS showed a weaker interaction (*K*_D_ = 3480 ng/mL). The weaker interaction of heparan sulfate with TSLP can be explained by its reduced number of negatively charged groups compared to heparin. In addition to their difference in molecular weight, heparin and heparan sulfate differ strongly in number of sulfate groups. While heparin bears approximately 1.4 to 2.5 sulfate groups per disaccharide unit, heparan sulfate contains just 0.55 to 1.25 sulfate groups per disaccharide unit.^33,48,49^ The weaker binding of heparin compared to the polyglycerol sulfates most likely results from the reduced density of sulfate groups, since carboxylate groups seem to be irrelevant for binding. While the lPGS_10(90%)_ presents 60 sulfate groups, heparin displays 54 sulfate groups (considering an average of 1.95 sulfate groups per disaccharide) over an increased molecular weight of 15 kDa, leading to a notable reduction of sulfate density. An increased number of functional groups also increases the number of counterions released during the binding process, and the resulting increase in entropy is the driving force of electrostatic interaction.^50,51^ The previously described role of hydrophobic interactions involved in protein binding of heparin may also contribute to the strong binding action we observed between TSLP and heparin or heparan sulfate.^52^

PG itself did not show any binding, confirming that the binding interaction was derived from the polyanions’ sulfate groups rather than their PG backbones.

### Polyanions’ Effect on TSLP-Mediated T_H_2-Polarization of CD4^+^ T Cells

TSLP is known to prime CD4^+^ T cells toward type 2 T helper cells (T_H_2) characterized by increased IL-4 and IL-13 release.^9,10^ After interaction of TSLP with its receptor (TSLPR), the resulting complex recruits the IL-7 receptor chain α. This recruitment leads in turn to phosphorylation of transcription factors, mainly STAT6 in CD4^+^ lymphocytes, which in turn promotes the expression of IL-4 and IL-13 (Figure 2C).^11^

**Figure 2:**
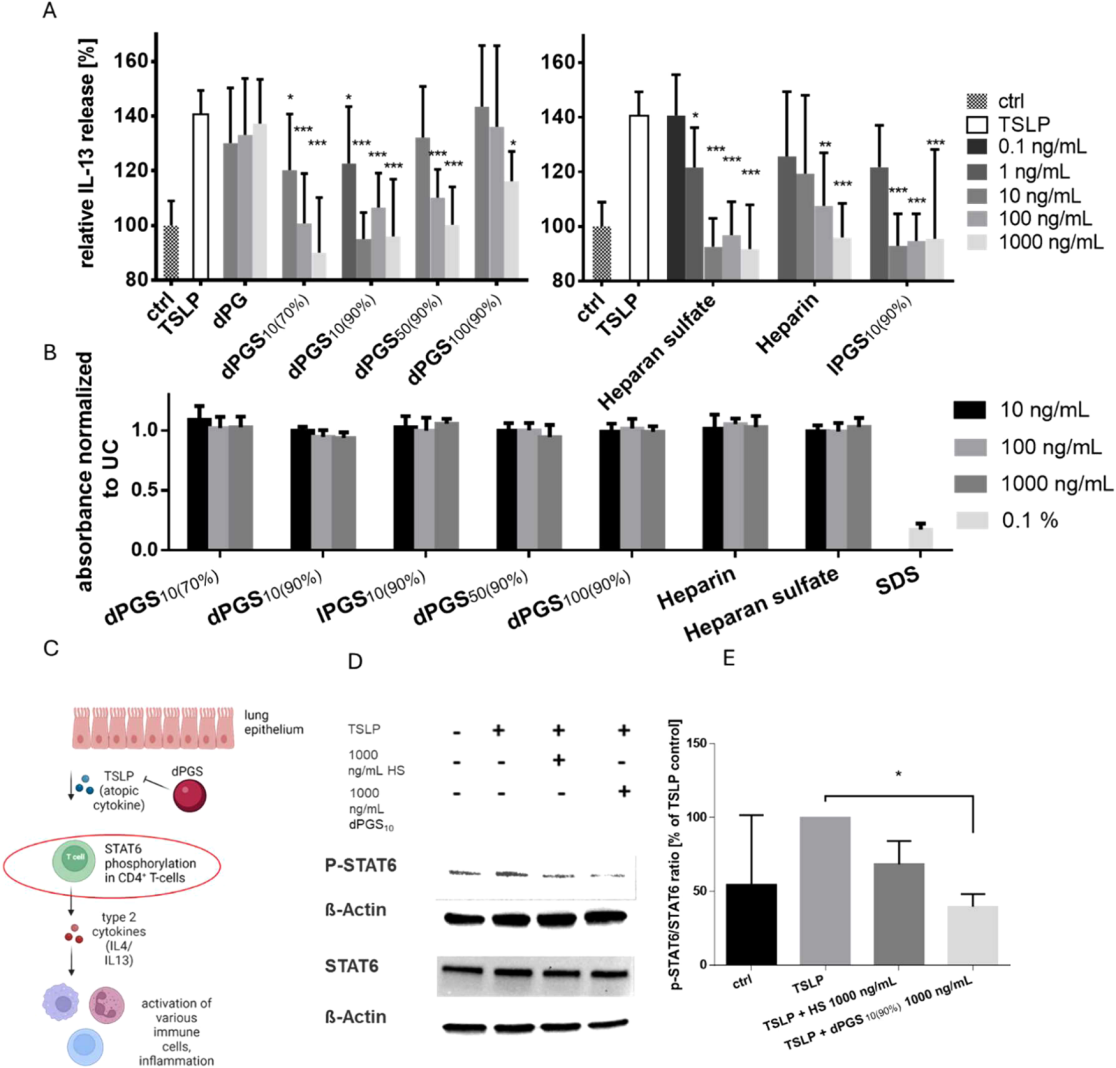
(A) The TSLP-triggered increase of IL-13 release in CD4^+^ T cells is significantly downregulated by all sulfated polyanions. dPG was used as a noncharged control. Measurements were performed in triplicate (*n* = 9). (B) Sulfated polymers do not affect cell viability of CD4^+^ T-cells. A CCK-8 assay was run after 24 h incubation. (C) Scheme of TSLP signaling pathway (created with BioRender). (D) TSLP induces STAT6 phosphorylation in CD4^+^ T cells, while dPGS_10(90%)_ significantly reduces STAT6 phosphorylation (Western blot). (E) Quantification of signal intensities shown in (D). Phosphorylation state was normalized to the TSLP-triggered control (*n* = 3). Statistical analyses were performed using one-way ANOVA in (A) and the Friedman test (due to small sample size) in (E), *P ≤ 0.05; **P ≤ 0.01; ***P ≤ 0.001. All data are presented as mean + SD.

We therefore used a CD4^+^ T cell assay to investigate the IL-13 release and thereby determine whether the strong electrostatic interactions influence the TSLP-mediated type 2 polarization of CD4^+^ T cells. (Before doing so, we used a cell viability assay (CCK8) to confirm that the investigated polyanions have no toxic effect on the T cells.)

None of the polymers showed toxicity at the tested concentrations (Figure 2B). Remarkably, all the polyglycerol sulfates significantly reduced the release of IL-13 from CD4^+^ lymphocytes (Figure 2A). Here, dPGS_10(90%)_, the smallest and highly sulfated polyglycerol, showed the most potent effect, leading to a significant IL-13 reduction at 1 ng/mL (*p* < 0.0.5). Other polymers required higher concentrations to significantly reduce IL-13 release: 10 ng/mL for lPGS_10(90%)_, 100 ng/mL for dPGS_50(90%)_, and 1000 ng/mL for dPGS_100(90%)_. The potent anti-inflammatory effect we observed is in accordance with the strong binding of polyglycerol sulfates to TSLP via electrostatic interactions as shown in Figure 1. To confirm that the reduced IL-13 secretion is attributable to interaction of TSLP and polyglycerol sulfate, we performed additional CD4^+^ T-cell activation assays via PMA/Ionomycin or bead-conjugated CD3/CD28 antibodies in the absence of TSLP. Here, adding 100 ng/mL of dPGS_10(90%)_ had no effect on the IL-13 release (SI Figure 8), which is in line with our hypothesis that the observed reduction of IL-13 release observed in Figure 2 is most likely attributable to dPGS-scavenging of TSLP and not other unspecific immunosuppressive effects. Interestingly, HS also led to a significant IL-13 reduction at 1 ng/mL.

Previous studies using selective STAT inhibitors have demonstrated that the TSLP-mediated type 2 response of CD4^+^ T cells is driven mainly by STAT6 phosphorylation (Figure 2C). To assess whether the IL-13 reduction by HS and dPGS_10(90%)_ was due to diminished TSLP–TSLPR interaction, we investigated the phosphorylation state of STAT6. A decrease in STAT6 phosphorylation would be expected in the event of reduced IL-13 release caused by TSLP inhibition.^11^ The quantification of STAT6 phosphorylation by Western blot shows a significant reduction (*p* < 0.05) of the phosphorylation state when adding dPGS_10(90%)_ (Figure 2D,E) and a less pronounced reduction of the phosphorylation state when adding HS. The dPGS_10(90%)_-mediated pSTAT6 reduction is in line with the observed strong binding to TSLP. A possible explanation for the less pronounced pSTAT6 reduction by HS could be that it requires long periods of time to interact strongly enough with TSLP, and then forms highly stable complexes that cause the observed potent reduction of IL-13 after 36 h. This slow but highly stable complex formation was also observed in SPR studies. Here, the *k*_a_ was relatively weak, but the *k*_d_ was comparable to that of the polyglycerol sulfates (see Table 2). Thus, the brief 20 min incubation time in the investigation of STAT6 phosphorylation may not have been long enough to enable fully realized HS-TSLP interaction.

An alternate explanation could be the interaction of HS with other cytokines. For example, heparan sulfate is known to bind IL-8 and might also influence T cell polarization in this way.^53^ Additionally, heparin and heparan sulfates are known to interact with IL-7, a cytokine structurally similar to TSLP, and were confirmed in one study to play a role in lymphoid cell binding by initiating binding to the surface.^54^ In that study, removing HS from the surface or adding heparin to the media significantly reduced IL-7 binding to cell surfaces. Further, the competition of heparin with cell-surface heparan sulfate for cytokine binding is the suspected reason for the curbed cytokine storm after application of low-molecular-weight heparin (LMWH) during SARS-COV-2 infection.^55^ IL-7 and IL-8 are only two examples of the wide range of heparin- and HS-binding proteins.^56^

Based on the obtained results, we became interested whether the polyanions also interact with other pro-inflammatory mediators relevant for type 2 inflammation and atopic diseases. Our own recent research has shown that FN occurs at elevated levels in the plasma of children with atopic dermatitis and can also trigger an atopic-like inflammation in human-based 3D bronchial tissue models.^44^ We therefore proceeded to investigate the affinity of the polyanions for FN.

For the analysis, we established a competitive assay based on the polyglycerol anions’ ability to disrupt the binding of FN to immobilized heparin (Figure 3A,B). We chose this assay setup instead of a direct binding assay due to the FN degradation that we had observed after attempting its covalent immobilization. Based on the relative binding values, we calculated the respective half-maximal inhibitory concentrations (IC_50_). The results in Table 3 show that lPGS_10(90%)_ and dPGS_10(90%)_ strongly bind FN, yielding an IC_50_ in the picomolar range (170 pM for dPGS_10(90%)_ and 280 pM for lPGS_10(90%)_). A reduction in the degree of sulfation from 90 to 40% led to a dramatic increase of the IC_50_ by more than 3 orders of magnitude to 960 nM, highlighting the need for high functionalization to realize strong electrostatic interactions. Again, in contrast to sulfation, the carboxylated compounds lPGC_10(90%)_ and dPGC_10(90%)_ showed no target binding up to 10 μM. Fibronectin interaction likely relies on binding of heparin to binding sites of type III domains, which are known to display several lysine and arginine amino acids that can form salt bridges with polyanions.^57^

**Table 3:** Inhibitory Potential of Sulfated and Carboxylated Linear and Dendritic Polyglycerols

Polyanion	*M_n_* (kDa)	IC_50_ (nM)^a^	IC_50_ (ng/mL)
dPGS_10(90%)_	9.9	0.17	1.70
lPGS_10(90%)_	9.9	0.29	2.90
dPGS_7(40%)_	7	960	67,200
dPGC_10(90%)_	10	no inhibition	
lPGC_10(90%)_	10	no inhibition	
dPG	5	no inhibition	

a Half-maximal inhibitory concentration (IC_50_) determined by competitive SPR assay.

**Figure 3:**
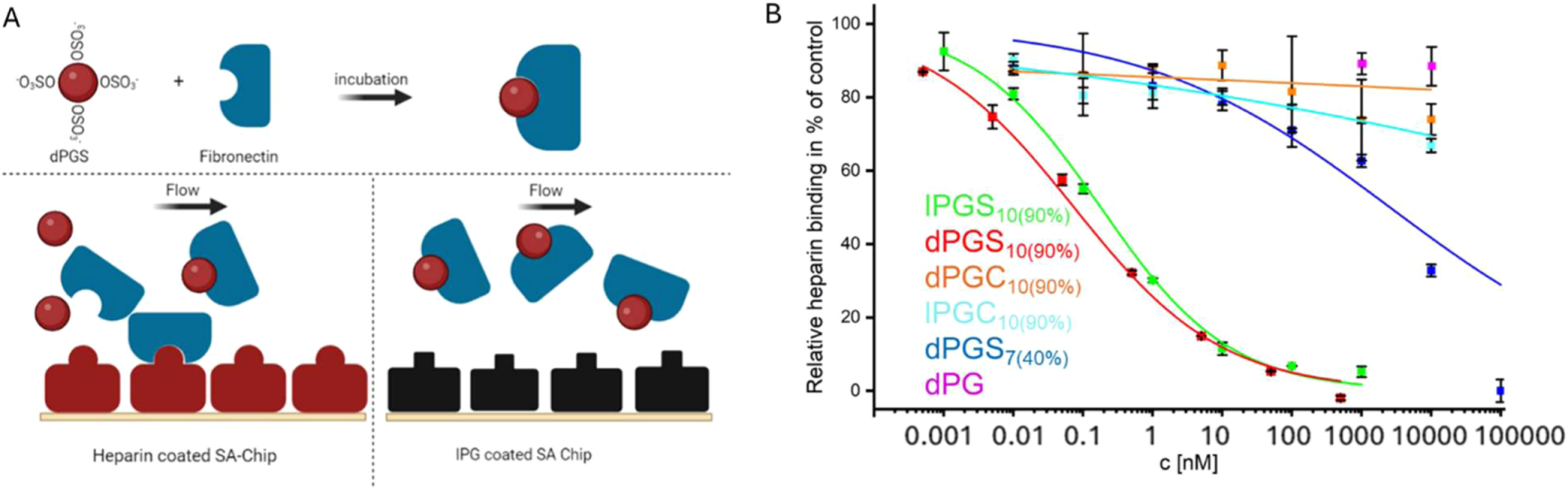
(A) Scheme showing the competitive SPR assay to determine polyanion-mediated inhibition of FN binding to immobilized heparin. Scheme was created with BioRender. (B) Concentration-dependent inhibition of FN binding to heparin by different linear and dendritic polyglycerol anions. The 100% value refers to the untreated control, and the inhibitor concentration at 50% binding results in the IC_50_ value. Measurements were performed in duplicate. All data are presented as mean + SD.

To expand our understanding of the evident inhibition activity into more physiological conditions, we next investigated the effect of the strongest inhibitor, dPGS_10(90%)_, on a human-based 3D inflamed bronchial epithelial tissue model.^44^ The decision to use a human-cell-based model instead of animal models was driven by the missing cross-reactivity between human and murine TSLP (only a 43% homology) and TSLPR (35%).^58^ Originally developed following the discovery of elevated FN levels in atopic infant plasma, the model we used was designed to prove whether FN induces an inflammatory phenotype and contributes to the atopic march. Although disease progression in humans typically takes years, a significant FN contribution to inflammation was observed in the model after a three-week cultivation. Here, the inflammation was triggered by adding 1 μg/mL of human plasma FN, leading to increased TSLP expression (Figure 4A–C) as well as a significant increase of IL-6 (Figure 4D). dPGS_10(90%)_ led to a dose-dependent reduction of TSLP expression at 10 μg/mL, as well as a significant reduction of IL-6 in the supernatant at 100 μg/mL. This effect can be explained by the strong binding of dPGS to FN, which likely reduces FN’s interaction with cell-surface integrins, and the FN-mediated NFκBp65 activation.^59^ It is noteworthy that the concentrations necessary to mediate anti-inflammatory effects in cell-based assays are significantly higher than the IC_50_ observed in the simplified binding assays. This disparity might result from nonspecific binding of dPGS_10(90%)_ to other proteins and to the collagen matrix of the bronchial epithelium model.

**Figure 4:**
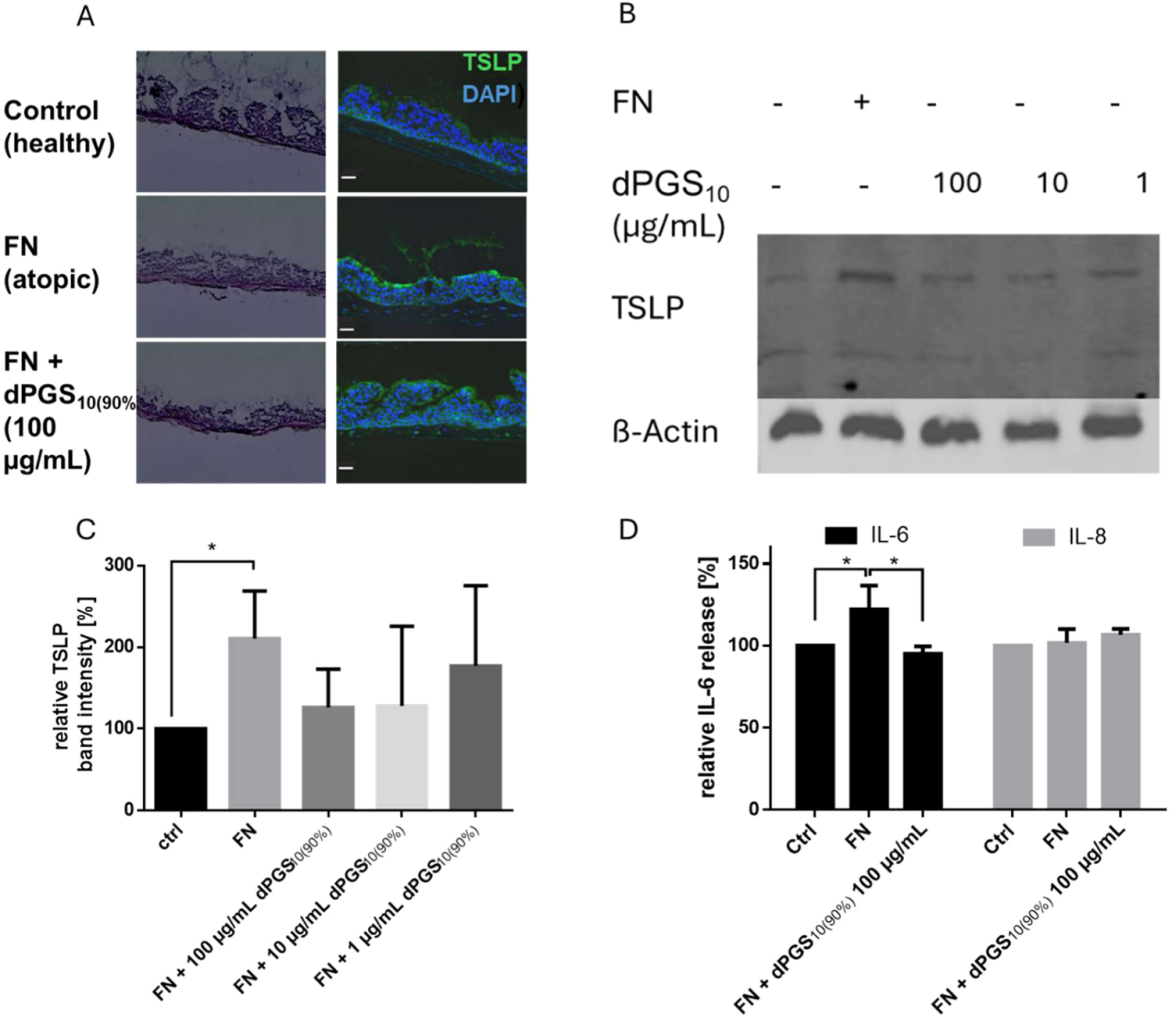
(A) dPGS_10(90%)_ reduces FN-induced TSLP expression. Cryosections of hematoxylin and eosin (H&E)-stained and immunofluorescence-stained tissues from healthy, inflamed and dPGS-treated atopic lung models. DAPI-stained nuclei are rendered in blue; TSLP is in green. Scale bar: 50 μm. (B) Representative Western blot showing TSLP expression levels after treatment of atopic lung models with 1, 10, and 100 μg/mL of dPGS_10(90%)_ (*n* = 3). (C) TSLP Western blot band intensity of lysed lung model, quantified by ImageJ and normalized to the intensity of the control. (D) dPGS_10(90%)_ reduces IL-6 level while leaving IL-8 expression unaffected in a bronchial epithelial tissue model triggered with FN. Cytokine release was determined by ELISA and normalized to the control of the individual donor (*n* = 3). Statistical analysis was performed using Friedman test, *P ≤ 0.05. All data are presented as mean ± SD.

In addition to binding with FN, dPGS is likely to interact with other cytokines that induce TSLP release, due to these cytokines’ positive surface charge, especially at high concentrations like those we investigated. A broad range of targets has previously been described for polyanions like dPGS and heparins.^60–62^ The previously observed reduction in neutrophil extravasation in the presence of dPGS, primarily attributed to L- and P-selectin inhibition, may also result partially from FN inhibition, given FN’s role as an adhesion molecule in immune cell migration.^27,63^ Inhibition of inflammation by FN signal disruption has been observed in murine models of vascular inflammation.^64^ Finally, FN is also known to regulate the binding of IL-7 to the cell surface by different stretching of the proteoglycan, an effect that might also be altered by dPGS binding.^65^

## Conclusions

In the study presented here, we discovered strong interactions of polyglycerol sulfates with the type 2 inflammation-driving factors thymic stromal lymphopoietin (TSLP) and fibronectin (FN), leading to anti-inflammatory effects on the TSLP-mediated T_H_2- polarization of CD4^+^ T cells and on the FN-mediated inflammation in inflamed bronchial epithelium models. A detailed analysis of the polyglycerol anions’ binding behavior suggested that polymer architecture is not essential to its binding to FN, while linear polymers can bind to TSLP more effectively than their dendritic counterparts. The inquiry into the impact of polyglycerol sulfates’ size on their affinity to TSLP showed only minor effects, with dPGS_10(90%)_ and dPGS_50(90%)_ showing the strongest affinity, while dPGS_100(90%)_ exhibited slightly reduced binding. Sulfate groups, on the other hand, proved essential to the polyanions’ interaction with both proteins; functionalization with carboxylate groups prevented significant interaction from occurring. This result echoes previous findings on the essential role of sulfates in binding to heparin binding proteins, in particular P- and L-selectin.^27^ Having established the importance of sulfate groups, we found that a high degree of sulfate functionalization was necessary: reducing the functionalization degree from 90 to 40% led to a 5000-fold weaker binding to FN, confirming charge–charge interactions as the driving force. Previous literature also describes a role for hydrophobic and hydrogen-bonding interactions in protein binding of heparin, an effect that could also be considered in the development of further specialized TSLP or FN binders. For example, hydrogen bonding plays a major role in the binding of heparin to brain natriuretic peptide and nonionic interactions are responsible for 60% of the free binding energy in binding to ATIII. Here, phenylalanine 121 and 122 are essential for the conformational change of the protein responsible for the stable complex formation after interaction with heparin.^52,66,67^

In addition, HS significantly reduced IL-13 at concentrations as low as 1 ng/mL. This high potency was initially surprising, since heparin showed markedly stronger interaction with TSLP than HS in the binding assay and a lower potency of IL-13 reduction. We believe that this result is attributable to the highly stable complexes formed between TSLP and HS, since its dissociation constant *k*_d_ is an order of magnitude smaller than that of TSLP to heparin (Table 2). The interaction of HS or heparin with other proteins is also likely, since both are known to bind to numerous additional proteins.

We also observed an anti-inflammatory effect for dPGS_10(90%)_ at a concentration of 10 μg/mL in the inflamed bronchial epithelium model. We believe that this effect results from the strong affinity of dPGS_10(90%)_ to FN, which we observed by SPR measurements. As this concentration of dPGS_10(90%)_ already exhibits significant anticoagulant activity, further optimization of the polymers would be necessary to target FN in type 2 inflammation.^68^ In contrast, the potent effects of heparin, HS and polyglycerol sulfates on T cells arise in the ng/mL range, and the concentrations necessary for IL-13 reduction are comparable to those required for the novel small-molecule TSLPR inhibitor BP79, with its IC_50_ of 23 ng/mL for IL-13 reduction in CD4^+^ T-cells.^11^ The current clinically approved inhibitors of type 2 inflammation are JAK1 and JAK2 inhibitors, glucocorticoids and monoclonal antibodies targeting TSLP, IL5, IL-4/IL-13Ra, and IgE.^21,69–72^ While these drug classes offer a variety of therapy options for T_H_2-driven inflammation, further options are still desired due to the high prevalence of T_H_2-driven inflammatory conditions, high production cost of antibodies, and side effects associated with JAK inhibitors and glucocorticoids.^11^

These synthetic polyglycerol sulfates present a new approach to dampening type 2 inflammation, joining the array of previously observed effects of polyglycerol sulfates, like reduced extravasation of leucocytes by interaction with L- and P-selectin and reduced complement system activation.^26,27,29^

In contrast to heparin and HS, polyglycerol sulfates have markedly reduced anticoagulant activity and also carry the advantage of tunable pharmacokinetics. While HS and heparin are rapidly excreted, polyglycerols have a longer half-life, which can also be adjusted by the inclusion of cleavable moieties like esters or ketals.^73–75^ The general approach of using macromolecules like heparin, HS, or polyglycerol sulfates to interact with heparin binding sites may be a viable alternative to small molecules, since these patches often occupy a large surface area of 300–600 Å^2^, an area challenging for a small molecule to effectively cover.^19,76^

Considering polyanions’ generally high likelihood of binding to a variety of proteins, more selective targeting of polyanions should be developed. For instance, we recently engineered a highly specific polycationic group for the binding of polyphosphate.^77^ A polyanionic counterpart could potentially enable highly specific binding of proinflammatory factors like TSLP or FN.

## Supplementary Material



## Notes

Polymer characterization, SPR experiments, uncropped Western blots (PDF)
