# Supplementary material for: Anti-Inflammatory Effects of Polyglycerol Sulfates and Natural Polyanions in Type 2 Inflammation
Source: Biomacromolecules. 2025 May 23;26(6):3819–29. doi: 10.1021/acs.biomac.5c00420 (PMC12152951; doi:10.1021/acs.biomac.5c00420)
Supplement: Supplementary file 1 [file bm5c00420_si_001.pdf]

Supporting Information for

Anti-inflammatory effects of polyglycerol sulfates and natural polyanions in type 2 inflammation

[†]Clemens Krage, [‡]Peyman Malek Mohammadi Nouri, [δ]Jens Dernedde [‡,$,#]Jayachandran N. Kizhakkedathu, [§,$,‡,ζ]Sarah Hedtrich, [†]Rainer Haag, [*†]Katharina Achazi*

[†] Institute für Chemie und Biochemie, Freie Universität Berlin, Takustrasse 3, D-14195 Berlin, Germany

[‡] Centre for Blood Research, Life Sciences Institute, University of British Columbia, 2350 Health Sciences Mall, Life Sciences Centre, Vancouver, BC V6T 1Z3, Canada

[δ]Institut für Laboratoriumsmedizin, klinische Chemie und Pathobiochemie, Charité-Universitätsmedizin Berlin, Augustenburger Platz 1, 13353 Berlin, Germany

[#]Department of Pathology and Laboratory Medicine, University of British Columbia, 2350 Health Sciences Mall, Life Sciences Centre, Vancouver, BC V6T 1Z3, Canada

[$]The School of Biomedical Engineering, University of British Columbia, 2350 Health Sciences Mall, Life Sciences Centre, Vancouver, BC V6T 1Z3, Canada

[§] Faculty of Pharmaceutical Sciences, University of British Columbia, Vancouver, BC V6T 1Z3, Canada

[ζ] Berlin Institute of Health at Charité – Universitätsmedizin Berlin, Germany

Corresponding authors:
Rainer Haag – Department of Biology, Chemistry, Pharmacy, Freie Universität Berlin, E-Mail: haag@chemie.fu-berlin.de
Katharina Achazi - Department of Biology, Chemistry, Pharmacy, Freie Universität Berlin, E-Mail: katharina.achazi@fu-berlin.de

DPG

The average molecular weight of 10 kDa of dPG was prepared as previously reported with the improved method in our group.[1-3] ^{1}H NMR (500 MHz, D_2O, δ (ppm)): 0.83 (3H, backbone initiator), 1.32 (2H, backbone initiator), 3.35-4.04 (m, backbone repeating units).

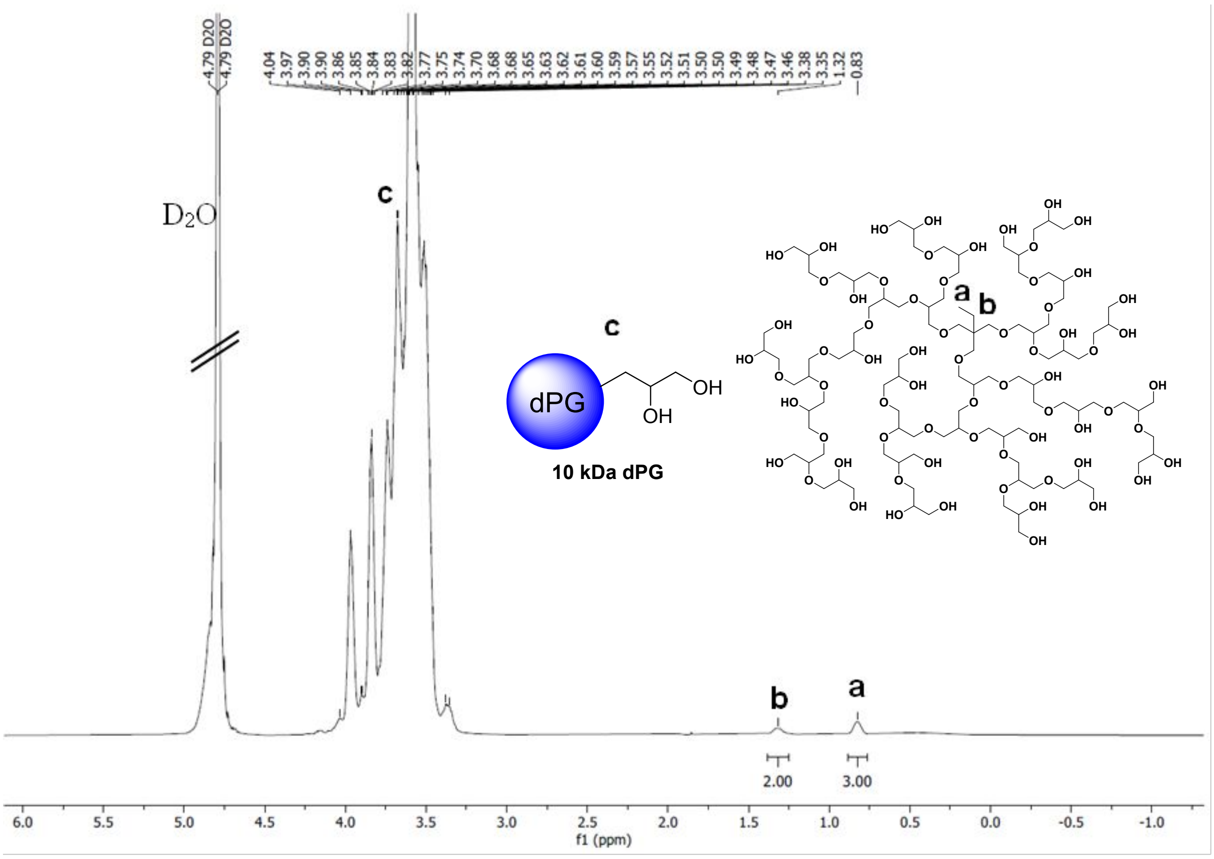

SI Figure 1: 1H NMR (500 MHz, D2O) of dendritic polyglycerol

dPGS:

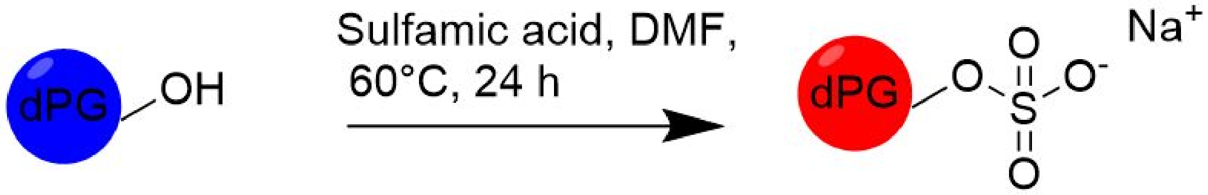

Scheme 1: sulfation of dendritic polyglycerol

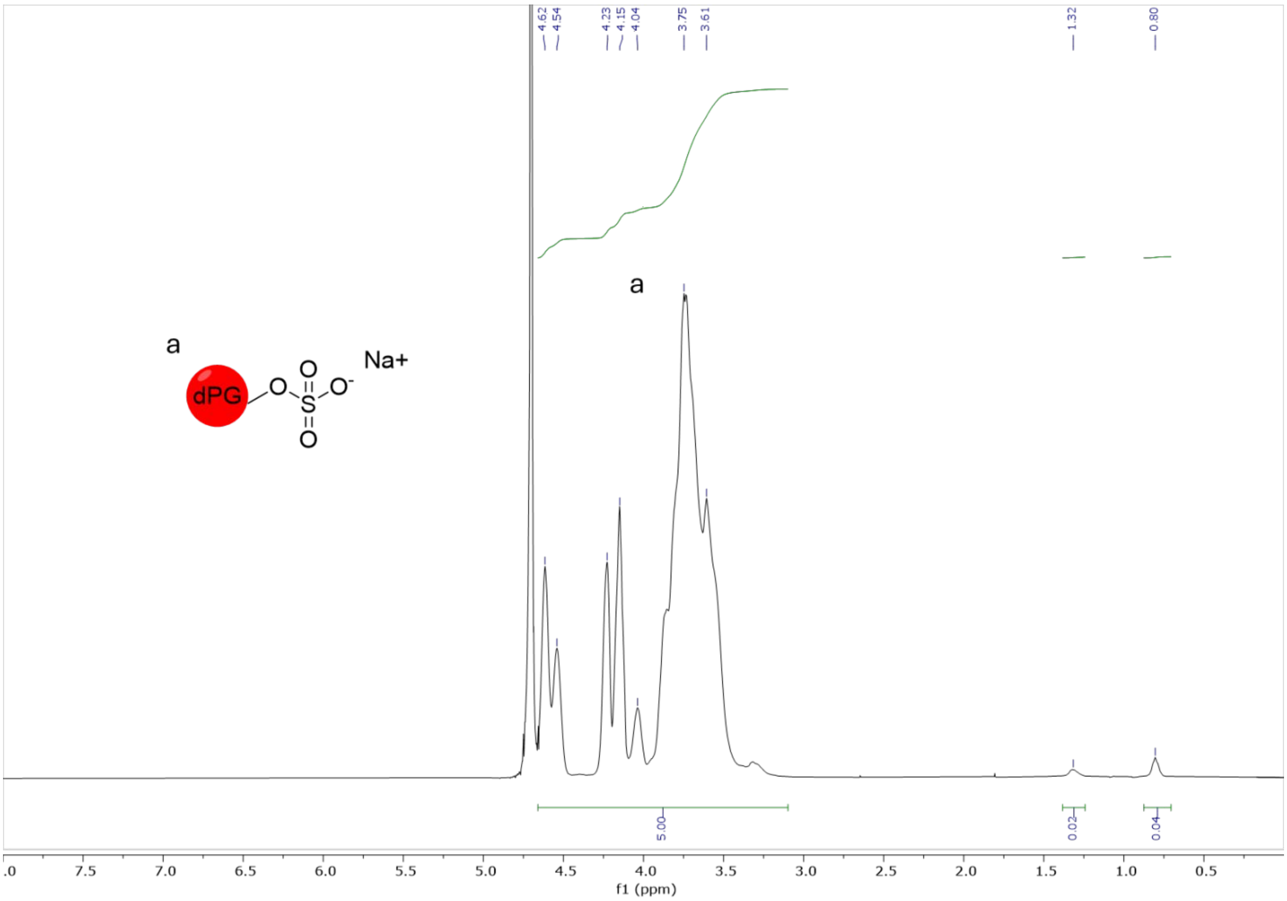

SI Figure 2: 1H NMR (500 MHz, D2O, δ)) of $dPGS_{10}$

Dried 5 kDa Polyglycerol (1 eq, 0.55 g, 0.11 mM) was dissolved in dimethylformamide (20 mL) and heated to 60˚C. Then, sulfamic acid (1.5 eq, 0.97 g, 10 mM) was added and the reaction was stirred for 24 h. The solution was then neutralized with NaOH and dialyzed against saturated NaCl solution and then water with 1 kDa cutoff tubes. After purification, the product solution was lyophilized. The number of sulfate groups was determined by elemental analysis. ^{1}H NMR (500 MHz, D_2O, δ (ppm)): 0.80 (3H, backbone initiator), 1.32 (2H, backbone initiator), 3.61-4.62 (m, backbone repeating units).

lPGS:

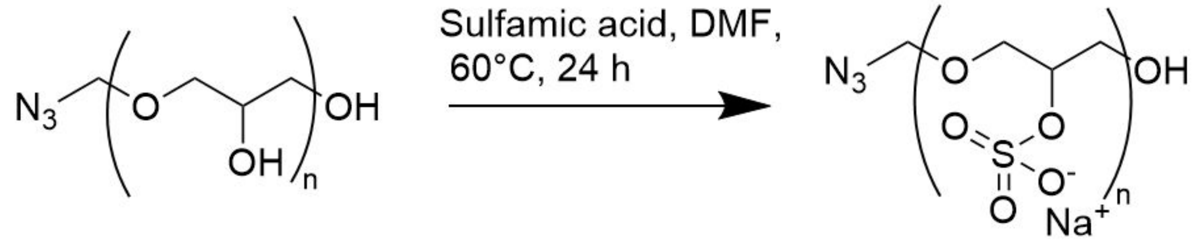

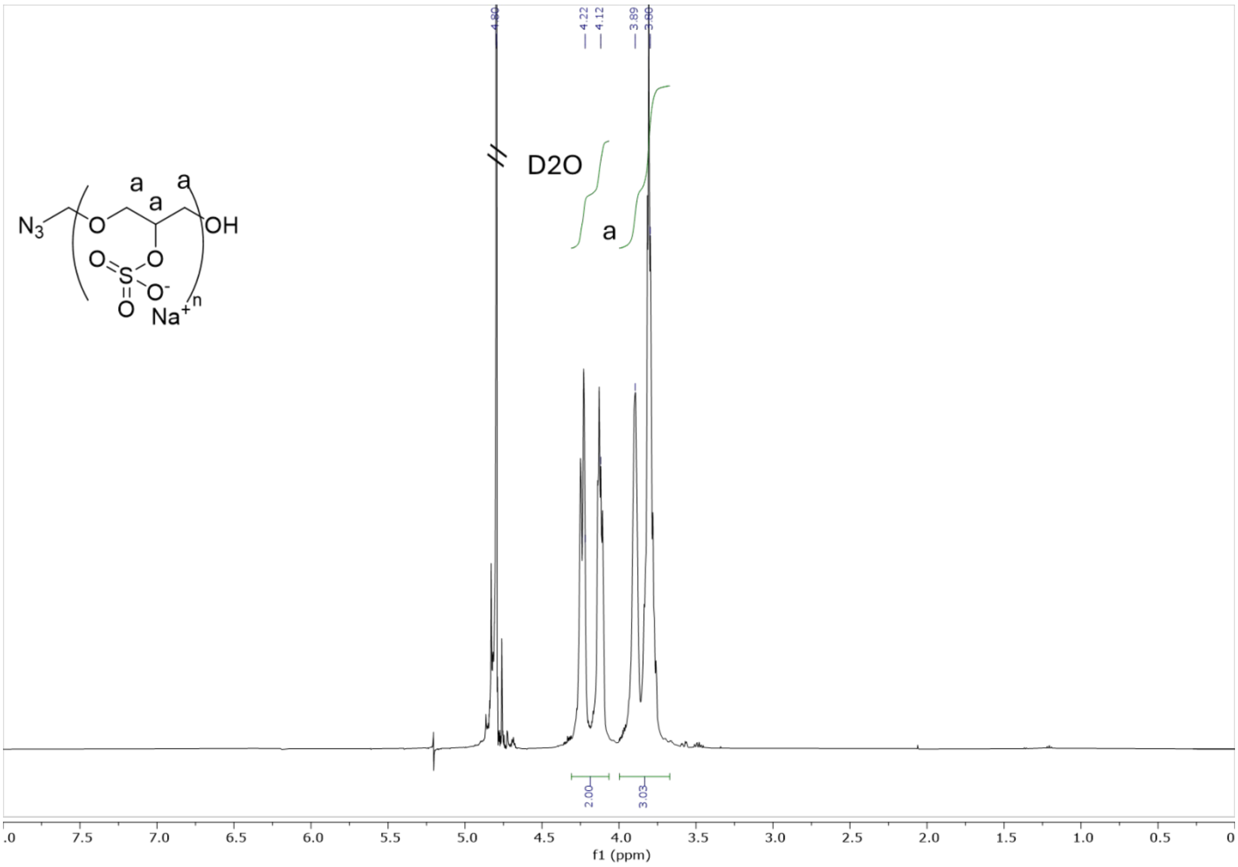

SI Figure 3: 1H NMR (500 MHz, D2O, δ (ppm)) of $lPGS_{10}$

Dried linear 5 kDa Polyglycerol (1 eq, 0.55 g, 0.11 mM) was dissolved in Dimethylformamide (20 mL) and heated to 60˚C. Then, sulfamic acid (1.5 eq, 0.97 g, 10 mM) was added and the reaction was stirred for 24 h. The solution was then neutralized with NaOH and dialyzed against saturated NaCl solution and then water with 1 kDa cutoff tubes. After purification, the product solution was lyophilized. The number of sulfate groups was determined by elemental analysis. ^{1}H NMR (500 MHz, D_2O, δ (ppm)): 3.80-4.22 (m, backbone repeating units).

dPGC:

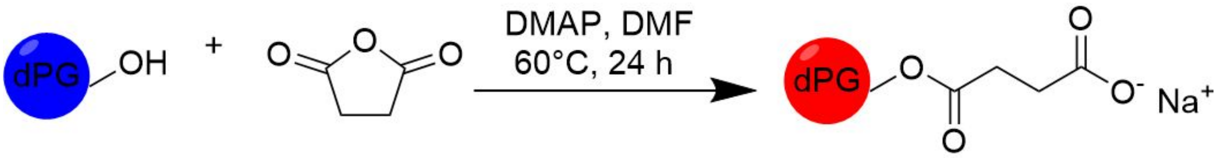

Scheme 2: carboxylation of dentritic polyglycerol

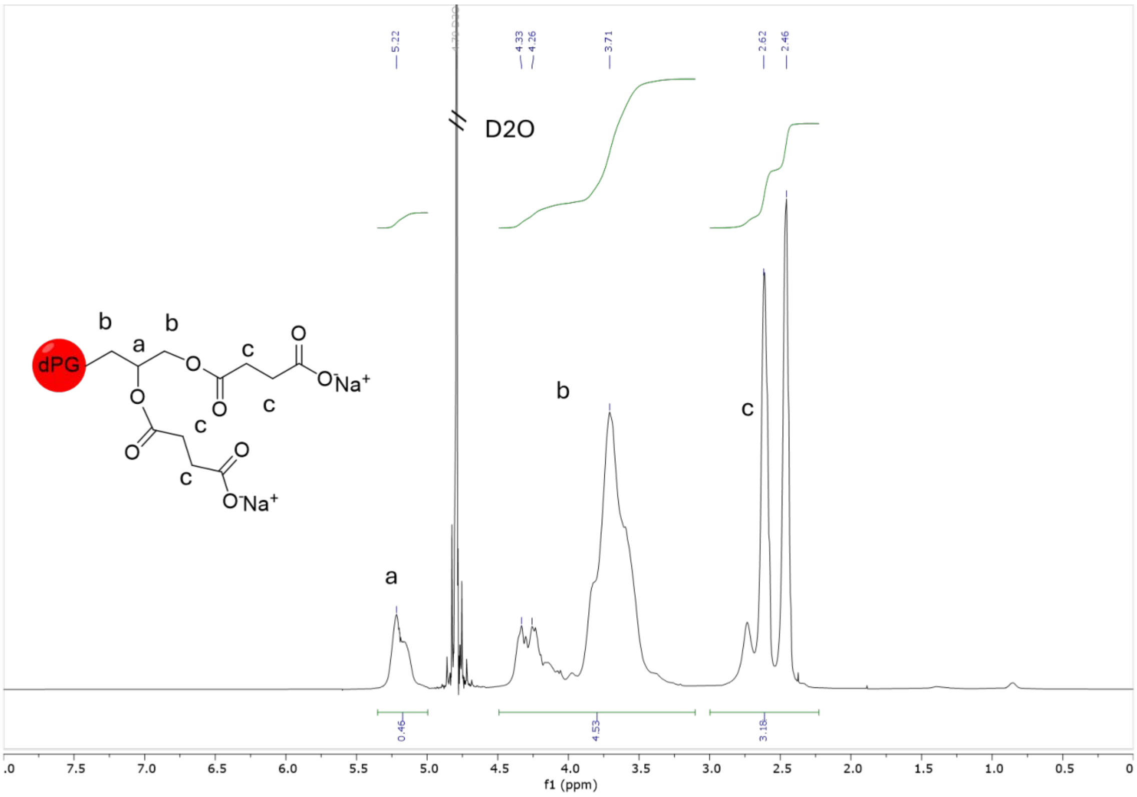

SI Figure 4: 1H NMR (500 MHz, D2O,) of $dPGC_{10}$

Dried 5 kDa Polyglycerol (1 eq, 0.45 g, 0.09 mM) was dissolved in Dimethylformamide (20 mL) and heated to 60˚C. Then, 4-(Dimethylamino)pyridine (1.2 eq, 0.892 g, 0.9 mM) and succinic anhydride (1.5 eq, 0.91 g, 9 mM) were added and the reaction was stirred for 24 h. The pH was adjusted to 9 with NaOH and the solution was dialyzed against saturated NaCl solution and then water with 1 kDa cutoff tubes. After purification, the product solution was lyophilized. The number of carboxylate groups was determined by ^{1}H NMR. ^{1}H NMR (500 MHz, D_2O, δ (ppm)): 2.46-2.62 (4H, succinic acid), 3.71-5.22 (m, backbone repeating units).

lPGC

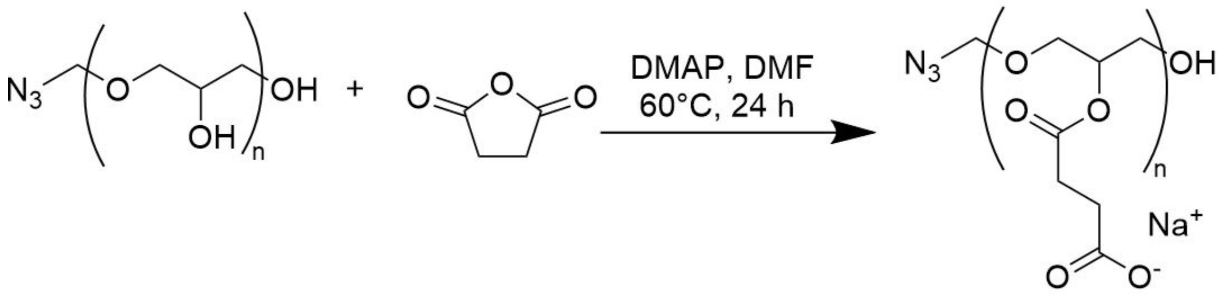

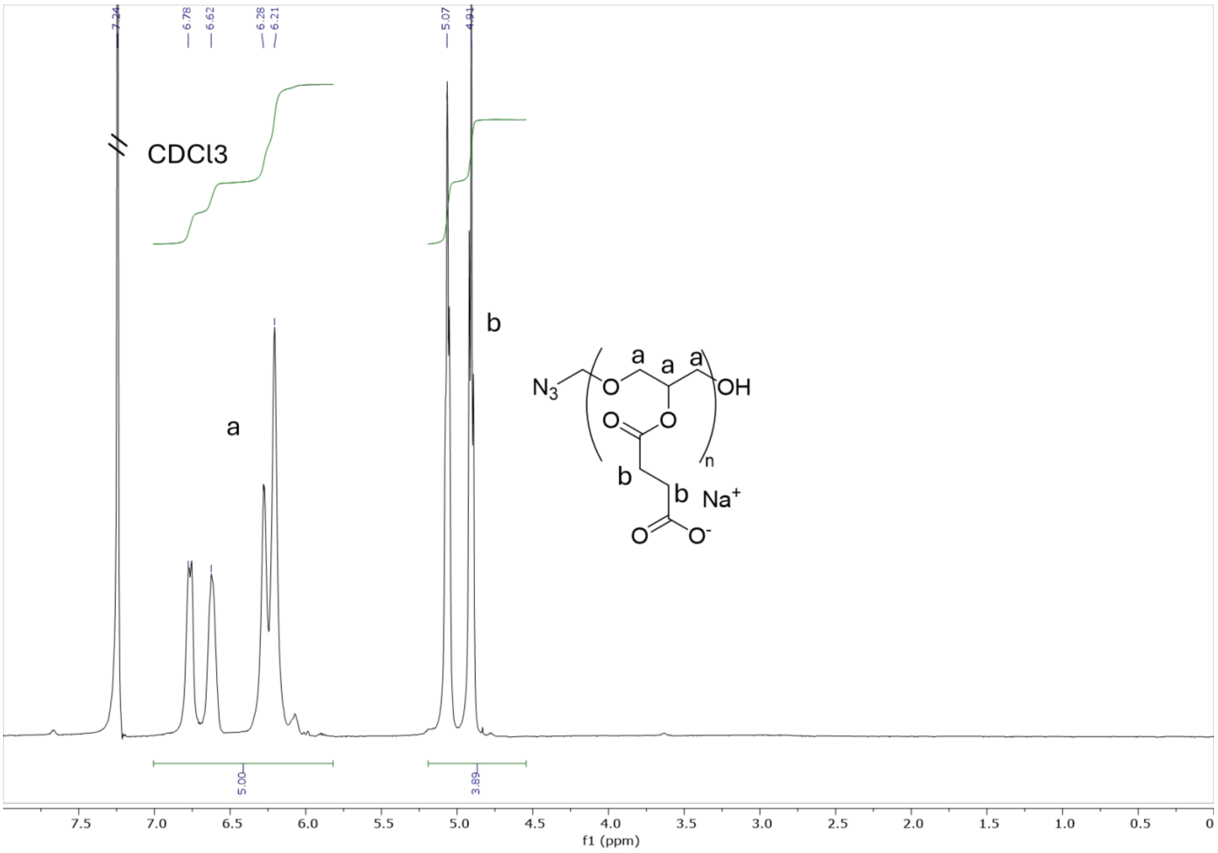

SI Figure 5: 1H NMR (500 MHz, D2O) of lPGC$_{10}$

Dried linear 5 kDa Polyglycerol (1 eq, 0.45 g, 0.09 mM) was dissolved in Dimethylformamide (20 mL) and heated to 60˚C. Then, 4-(Dimethylamino)pyridine (1.2 eq, 0.892 g, 0.9 mM) and succinic anhydride (1.5 eq, 0.91 g, 9 mM) were added and the reaction was stirred for 24 h. The pH was adjusted to 9 with NaOH and the solution was dialyzed against saturated NaCl solution and then water with 1 kDa cutoff tubes. After purification, the product solution was lyophilized. The number of carboxylate groups was determined by ^{1}H NMR. ^{1}H NMR (500 MHz, D_2O, δ (ppm)): 4.91-5.07 (4H, succinic acid), 6.21-6.78 (m, backbone repeating units).

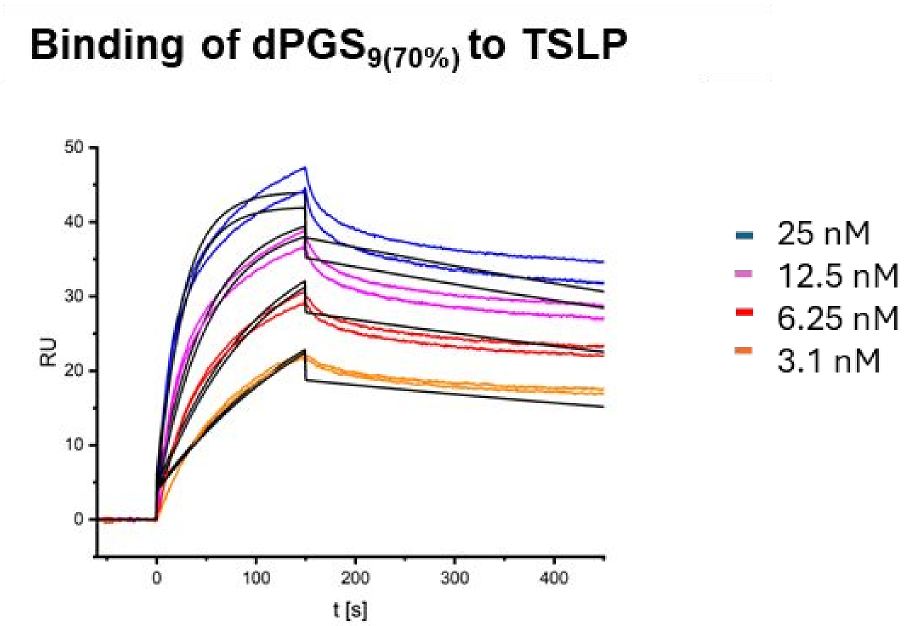

SI Figure 6: SPR sensorgram of $dPGS_{9(70\%)}$ binding to TSLP immobilized on an SA-Sensor, all concentrations were injected as duplicates, black lines represent the 1:1 kinetic fits.

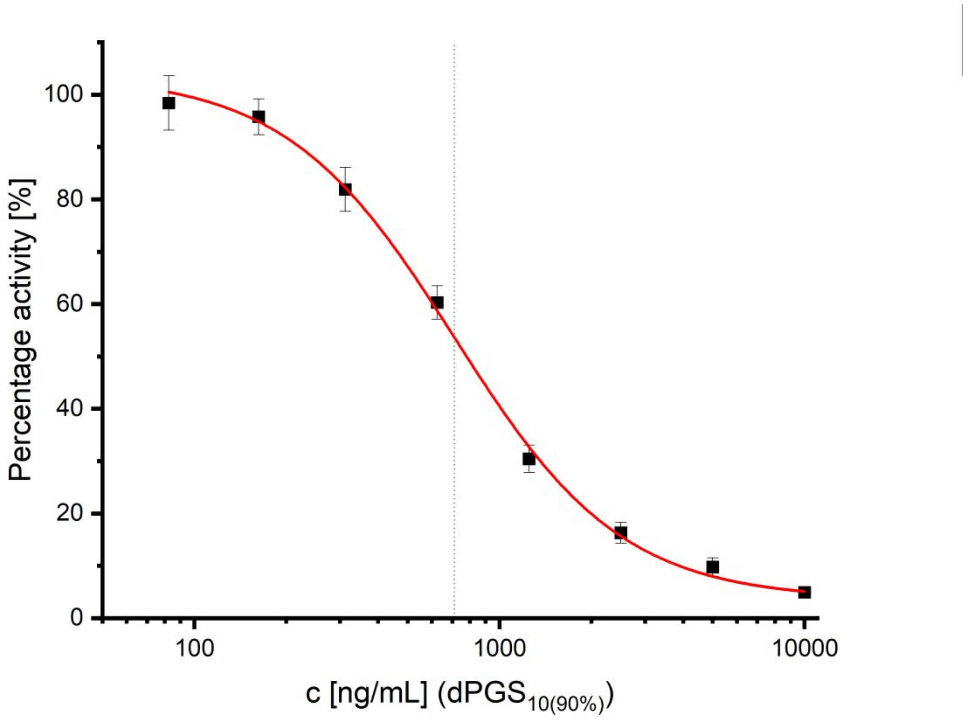

SI Figure 7: Competitive assay of TSLP binding to a TSLPR-IL7Rα fusion protein. 711 ng/mL of $dPGS_{10(90\%)}$ were necessary to reduce the binding of TSLP to TSLPR-IL7R fusion protein by 50%. All data represent mean + standard deviation.

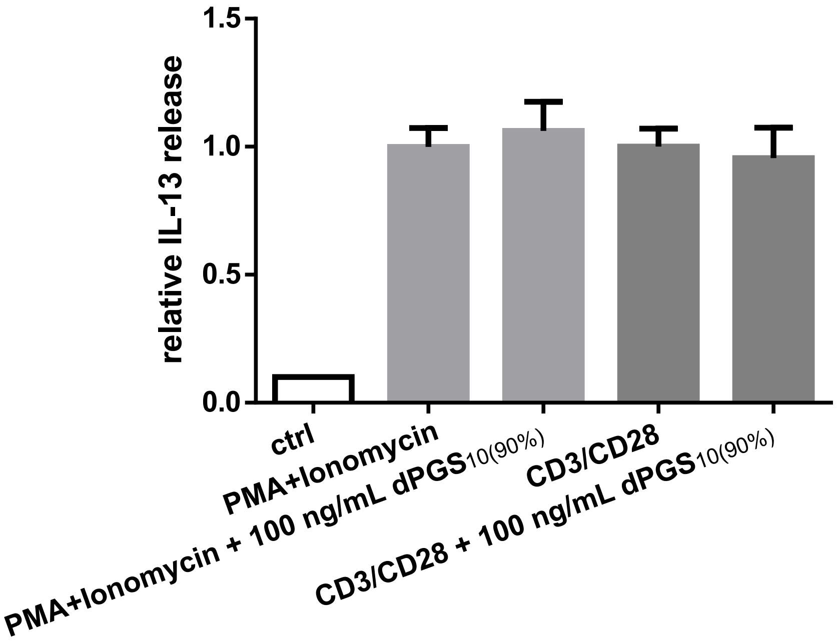

SI Figure 8: IL-13 release of CD4+ T-cells after 72 h activation via PMA+Ionomycin or via CD3/CD28-antibody conjugated beads in the absence of TSLP normalized to the activated control. Adding dPGS$_{10(90\%)}$ does not lead to a diminished IL-13 release in the absence of TSLP. The IL-13 concentration of the non-activated CD4+ T-cells was below the limit of detection (4 ng/mL), n = 6. All data represent mean + standard deviation.

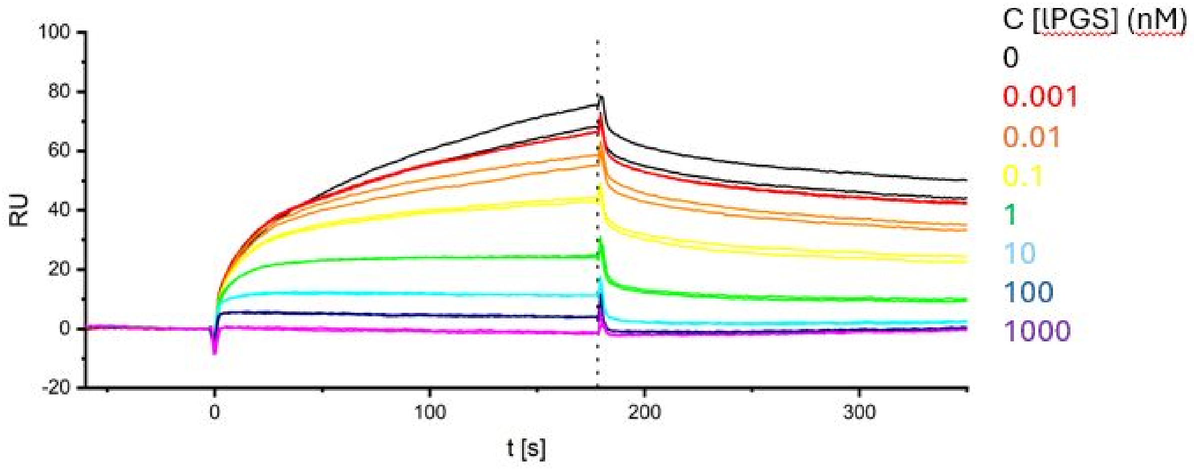

SI Figure 9: Representative SPR sensorgram of FN-Heparin inhibition Assay in presence of lPGS$_{10}$. Association time: 180 s, dissociation time 300 s.

Representative uncropped Western blots:

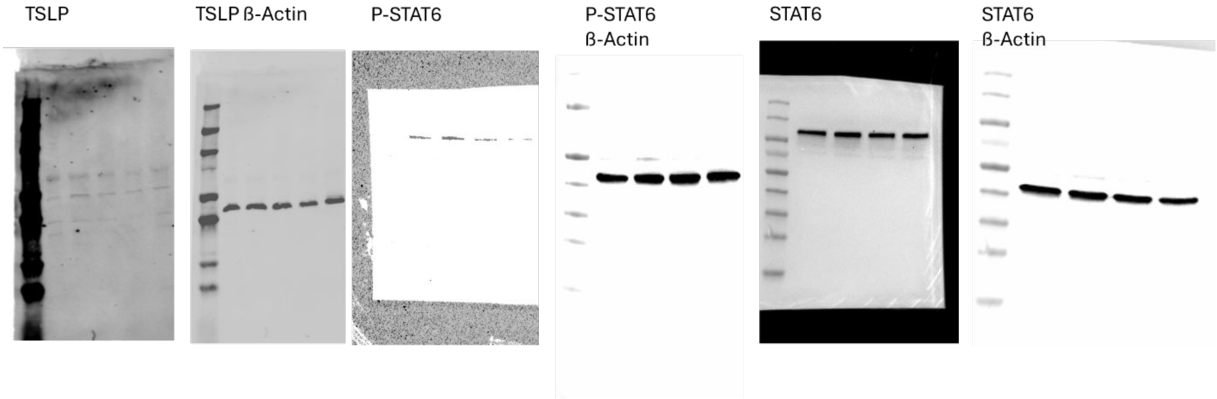

SI Figure 10: Representative uncropped western blots

References:

(1) Haag, R.; Sunder, A.; Stumbé, J. F. An approach to glycerol dendrimers and pseudo-dendritic polyglycerols. *J Am Chem Soc* **2000**, *122* (12), 2954-2955. DOI: DOI 10.1021/ja994363e.
(2) Sunder, A.; Hanselmann, R.; Frey, H.; Mülhaupt, R. Controlled synthesis of hyperbranched polyglycerols by ring-opening multibranching polymerization. *Macromolecules* **1999**, *32* (13), 4240-4246. DOI: DOI 10.1021/ma990090w.
(3) Wallert, M.; Plaschke, J.; Dimde, M.; Ahmadi, V.; Block, S.; Haag, R. Automated Solvent-Free Polymerization of Hyperbranched Polyglycerol with Tailored Molecular Weight by Online Torque Detection. *Macromol Mater Eng* **2021**, *306* (7). DOI: ARTN 2000688

10.1002/mame.202000688.
